# Prevalence of Curable Sexually Transmitted Infections in a Population-Representative Sample of Young Adults in a High HIV Incidence Area in South Africa

**DOI:** 10.1097/OLQ.0000000000001871

**Published:** 2023-10-08

**Authors:** Jana Jarolimova, Glory Chidumwa, Natsayi Chimbindi, Nonhlanhla Okesola, Jaco Dreyer, Theresa Smit, Janet Seeley, Guy Harling, Andrew Copas, Kathy Baisley, Maryam Shahmanesh, the Isisekelo Research Group, (Carina Herbst, Nuala McGrath, Thembelihle Zuma, Thandeka Khoza, Ngundu Behuhuma, Ingrid V. Bassett, Lorraine Sherr

**Affiliations:** From the ∗ Massachusetts General Hospital, Boston, MA; † Africa Health Research Institute, KwaZulu-Natal; ‡ Wits Reproductive Health & HIV Institute (Wits Health Consortium), University of the Witswatersrand, Johannesburg, South Africa; § University College London, London, United Kingdom; ¶ University of KwaZulu-Natal, Durban, South Africa; ∥ University of Southampton, Southampton; ∗∗ London School of Hygiene & Tropical Medicine, London, United Kingdom; †† University of the Witwatersrand, Johannesburg, South Africa

## Abstract

**Background:**

Recent population-representative estimates of sexually transmitted infection (STI) prevalence in high HIV burden areas in southern Africa are limited. We estimated the prevalence and associated factors of 3 STIs among adolescents and young adults (AYA) in rural South Africa.

**Methods:**

Between March 2020 and May 2021, a population-representative sample of AYA aged 16 to 29 years were randomly selected from a Health and Demographic Surveillance Site in rural KwaZulu-Natal, South Africa, for a 2 × 2 factorial randomized controlled trial. Participants in 2 intervention arms were offered baseline testing for gonorrhea, chlamydia, and trichomoniasis using GeneXpert. Prevalence estimates were weighted for participation bias, and logistic regression models were used to assess factors associated with STIs.

**Results:**

Of 2323 eligible AYA, 1743 (75%) enrolled in the trial. Among 863 eligible for STI testing, 814 (94%) provided specimens (median age of 21.8 years, 52% female, and 71% residing in rural areas). Population-weighted prevalence estimates were 5.0% (95% confidence interval [CI], 4.2%–5.8%) for gonorrhea, 17.9% (16.5%–19.3%) for chlamydia, 5.4% (4.6%–6.3%) for trichomoniasis, and 23.7% (22.2%–25.3%) for any STI. In multivariable models, female sex (adjusted odds ratio [aOR], 2.24; 95% CI, 1.48–3.09) and urban/periurban (vs. rural) residence (aOR, 1.48; 95% CI, 1.02–2.15) were associated with STIs; recent migration was associated with lower odds of STI (aOR, 0.37; 95% CI, 0.15–0.89). Among those with an STI, 53 (31.0%) were treated within 7 days; median time to treatment was 11 days (interquartile range, 6–77 days).

**Conclusions:**

We identified a high prevalence of curable STIs among AYA in rural South Africa. Improved access to STI testing to enable etiologic diagnosis and rapid treatment is needed.

## Abstract 2

Prevalence of gonorrhea, chlamydia, and trichomoniasis is high among adolescents and young adults in rural South Africa, and higher among women and those residing in urban/periurban areas.

Curable sexually transmitted infections (STIs) are common worldwide, with more than 1 million new cases of gonorrhea, chlamydia, trichomoniasis, or syphilis estimated to occur globally every day.^1^ When untreated, STIs can cause significant morbidity, particularly for women, leading to complications such as pelvic inflammatory disease, ectopic pregnancy, infertility, pregnancy complications, and newborn infection.^2,3^ Furthermore, STI-induced genital inflammation and genital HIV shedding can increase risks of HIV acquisition and transmission, even when the STI is asymptomatic.^4–6^ A majority of STIs occur in low- and middle-income countries, with the highest age-standardized incidence rates and a greatest number of disability-adjusted life years lost in sub-Saharan Africa.^7^ In southern Africa, there is strong epidemiologic overlap between curable STIs and HIV, particularly among adolescents and young adults, who are at highest risk for STI acquisition and have the highest HIV incidence rates.^7,8^ For these populations, improved diagnosis and treatment of curable STIs is key to reducing morbidity and is an important component of multimodal HIV prevention.

Because of a lack of accessible and affordable diagnostic testing, STIs in low- and middle-income countriess are predominantly managed using a syndromic approach.^9^ This approach misses a substantial proportion of STIs because they frequently remain asymptomatic.^10^ The World Health Organization (WHO) in the global health sector strategies for HIV, viral hepatitis, and STIs for 2022 to 2030 recommends a transition from syndromic to etiologic management of STIs and calls for increased screening of priority populations, including youth.^11^ The WHO has additionally recommended integration of STI care with other health services, including HIV prevention and treatment.^11^ However, screening and surveillance programs remain limited, and there are few recent population-representative data on STI prevalence to inform efforts at care integration.

In South Africa, which has among the highest HIV incidence and prevalence rates worldwide,^12^ STI prevalence is predicted to be high, with model-based prevalence estimates of 6.6% for gonorrhea and 14.7% for chlamydia among women and 3.5% gonorrhea and 6.0% chlamydia among men.^13^ Studies have found STI prevalence as high as 42% for chlamydia and 11% for gonorrhea among adolescent girls and young women in Cape Town.^14^ However, few population-representative studies of STI prevalence exist from areas of high HIV incidence in South Africa, particularly among both women and men, and previous prevalence data have not been recently updated.^15^ We aimed to use STI screening among a population-representative cohort of adolescents and young adults selected from a Health and Demographic Surveillance Site (HDSS) in rural KwaZulu-Natal, South Africa,^16^ to provide updated STI prevalence estimates among adolescents and young adults in this setting and assess for factors associated with having an STI.

## MATERIALS AND METHODS

### Study Setting

This study was conducted within the HDSS in uMkhanyakude district in rural KwaZulu-Natal, South Africa. Since 2000, the Africa Health Research Institute (AHRI; formerly Africa Centre for Health and Population Studies) has been conducting annual household-based surveys to collect data on births, deaths, demographics, and migration patterns. The HDSS was expanded in 2017 to cover 845 km^2^ with approximately 140,000 individuals in 20,000 households.^16^ The area has a high rate of unemployment (62% of adults without formal employment) and HIV prevalence of 19% among men and 40% among women aged 15 to 54 years.^16^

### Study Design

This study reports baseline data from a 2 × 2 factorial randomized controlled trial evaluating the acceptability, feasibility, and preliminary population-level impact of integrated sexual and reproductive health (SRH) services with or without peer support on the prevalence of transmissible HIV.^17^ The AHRI HDSS was used as a sampling frame to randomly select 3000 men and women aged 16 to 29 years, stratified by sex and area, to be assessed for eligibility. All eligible were approached for enrollment with a goal of at least 1500 eligible and enrolled participants. Men and women aged 16 to 29 years, residing in the HDSS area, willing and able to provide informed consent, and willing to be contacted at 12 months for HIV testing, were eligible to enroll in the trial. At enrollment, participants were randomized to 1 of 4 study arms: (*a*) enhanced standard of care (referral to adolescent and youth friendly services [AYFS] comprising clinic-based, nurse-led HIV testing with linkage to antiretroviral therapy [ART] or HIV preexposure prophylaxis), (*b*) SRH (home-based self-collection of STI specimens and referral to AYFS for integrated SRH and HIV testing), (*c*) peer support (referral to peer navigator to assess health, social, and educational needs and provide risk-informed HIV prevention and referral to AYFS),^18^ or (*d*) SRH and peer support. Participants randomized to the 2 SRH intervention arms were offered STI testing at study enrollment. Sample size for this analysis was determined by the total number of participants randomized to the SRH arms and providing specimens for STI testing.

### Study Procedures

After informed consent, participants randomized to either of the 2 SRH arms were offered home-based STI specimen collection. For female participants, research staff described the procedure to self-collect a vaginal swab. Menstruating females provided urine specimens. Male participants were instructed to collect a first-catch urine specimen. All participants were provided an AYFS clinic referral to receive their STI test results in 7 days. Participants were informed that if any test results return positive and they do not present to the clinic, research staff will attempt to contact them to ensure they receive treatment. Treatment of STI was provided according to South African national clinical guidelines (single-dose ceftriaxone and azithromycin for gonorrhea, single-dose azithromycin or 7-day course of doxycycline for chlamydia, single-dose metronidazole for trichomoniasis).^19^ Receipt of treatment was verified through AYFS clinic records and study documentation, participant self-report on follow-up contact, or documentation of failed contact attempts.

### Data collection

Sexually transmitted infection specimens were transported to the AHRI central laboratory in Durban. Testing for *Neisseria gonorrhoeae*, *Chlamydia trachomatis*, and *Trichomonas vaginalis* was conducted by real-time polymerase chain reaction by GeneXpert (Cepheid, Sunnyvale, CA). Valid STI test results were recorded as “detected” or “not detected.” Invalid test results were recorded as “invalid” or “error” based on test platform output. To minimize research procedures at enrollment to emulate real-world implementation of the interventions, study-specific questionnaires were not administered at the time of STI specimen collection. Sociodemographic data including education (years of completed education), employment (none, part-time, full-time), marital status (married, not married, informal union), household socioeconomic status (combined household asset index), and migration history (no migration, internal migration, in-migration, external migration) were derived from linking study participants to the annual HDSS household-level survey conducted in 2019.

### Statistical analysis

We summarized participants' demographic data using medians and interquartile ranges (IQRs) for continuous variables and frequency counts and percentages for categorical variables. Frequency counts and percentages with 95% confidence intervals (CIs) were calculated for the prevalence estimate of each individual STI and prevalence of any STI. To account for participation bias, we calculated weighted prevalence estimates to account for the stratified sample design, calculated as the inverse probability of study participation in strata defined by age group and sex. We used logistic regression to estimate the odds ratios and 95% CIs for factors associated with the presence of any curable STI and factors associated with treatment in univariate and multivariable models. Age and sex were included a priori in the multivariable model; other factors with *P* < 0.2 in univariate logistic regression were also included in the multivariable model; for treatment completion, age- and sex-adjusted models were used. Missing data were not imputed. All reported *P* values were 2-tailed; *P* < 0.05 was considered statistically significant. Analyses were conducted using Stata version 16.1 (Stata Corp, College Station, TX).

### Ethical considerations

The study protocol was approved by the Biomedical Research Ethics Committee of the University of KwaZulu-Natal (BREC/00000473/2019), the University College of London Research Ethics Committee (5672/003), and the Mass General Brigham Institutional Review Board (2021P002574). Written informed consent was obtained from all participants 18 years or older; verbal assent with written informed consent from a parent or guardian was obtained for all participants aged 16 to 17 years.

### Patient and public involvement

The peer support and sexual health intervention was co-created with young people in uMkhanyakude district and delivered by peers. Young people and the AHRI community advisory board were involved from research inception through to analysis. Study findings were shared with the research participants and their communities, as well as health officials and program implementers.

## RESULTS

**Figure 1:**
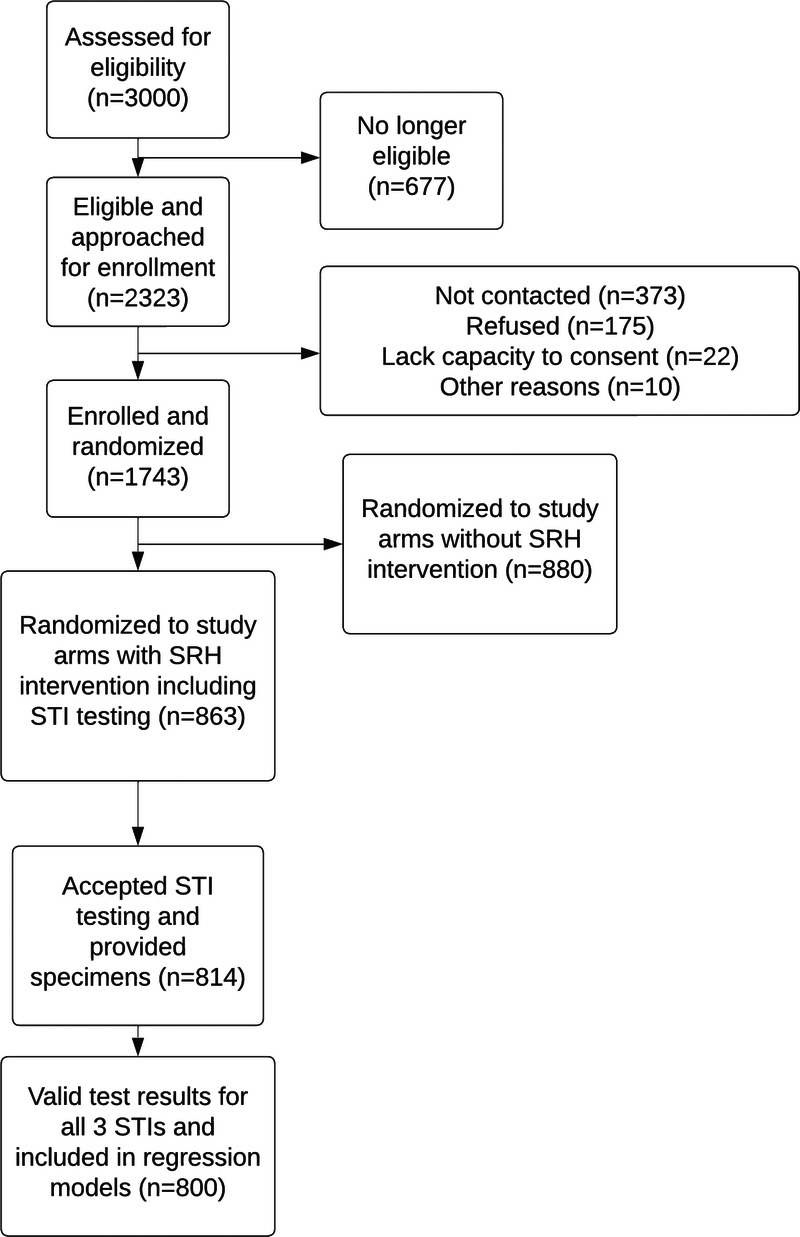
Flow diagram of study participants.

Between March 4, 2020, and May 24, 2021, 3000 adolescents and young adults were assessed for eligibility; 2323 were found to be eligible and were invited to participate, of whom 1743 (75%) enrolled in the randomized controlled trial (Fig. 1). Of these, 863 were randomized to the 2 study arms offering STI testing, and 814 (94%) accepted testing and provided specimens. There was no difference by sex between those who consented and did not consent to STI testing (*P* = 0.270). Among 427 female participants who provided specimens, 116 of 427 (27.2%) provided urine specimens; the remainder (311 of 427 [72.8%]) provided self-collected vaginal swab specimens. Among those tested for STIs, 52% were female, median age was 21.8 (IQR, 8.8–25.6) years, and 29% resided in urban or periurban areas. Additional participant demographics are presented in Table 1.

**TABLE 1:** Demographic Characteristics of Participants, by Sex

	Female (n = 427), n (%)	Male (n = 387), n (%)	Total (n = 814), n (%)
Age, median [IQR], y	22.5 [18.9–25.8]	21.2 [18.8–25.3]	21.8 [18.8–25.6]
Age category, y			
16–19	142 (33)	151 (39)	293 (36)
20–24	148 (35)	133 (34)	281 (35)
25–29	137 (32)	103 (27)	240 (29)
Highest level of education			
Some primary	13 (3)	14 (4)	27 (3)
Some secondary	183 (43)	211 (55)	394 (48)
Matric or above	178 (42)	116 (30)	294 (36)
Missing	53 (12)	46 (12)	99 (12)
Employment*			
Employed	25 (6)	38 (10)	63 (8)
Not employed	256 (60)	200 (52)	456 (56)
Missing	146 (34)	149 (39)	295 (36)
Marital status^†^			
Not married	98 (23)	123 (32)	221 (27)
Married or informal union	216 (51)	131 (34)	347 (43)
Missing	113 (26)	133 (34)	246 (30)
Socioeconomic status, tertiles			
Low	145 (34)	114 (29)	259 (32)
Middle	140 (33)	121 (31)	261 (32)
High	123 (29)	133 (34)	256 (31)
Missing	19 (4)	19 (5)	38 (5)
Residence			
Rural	296 (69)	281 (73)	577 (71)
Urban or periurban	130 (30)	105 (27)	235 (29)
Missing	1 (0.2)	1 (0.2)	2 (0.25)
Migration in preceding 2 y^‡^			
Never	368 (86)	331 (86)	699 (86)
Internal migration	2 (0.5)	3 (1)	5 (1)
External migration	27 (6)	26 (7)	53 (7)
Missing	30 (7)	27 (7)	57 (7)

*Employed = full-time and part-time employed. Employment not reported for the majority of participants 18 years or younger.

^†^Only 5 participants reported as “married.” Marital status not reported for the majority of participants 18 years or younger.

^‡^In the 2 years preceding date of STI testing. Internal migration is migration within the HDSS area. External migration includes participants who migrated into or outside of the HDSS area.

**Figure 2:**
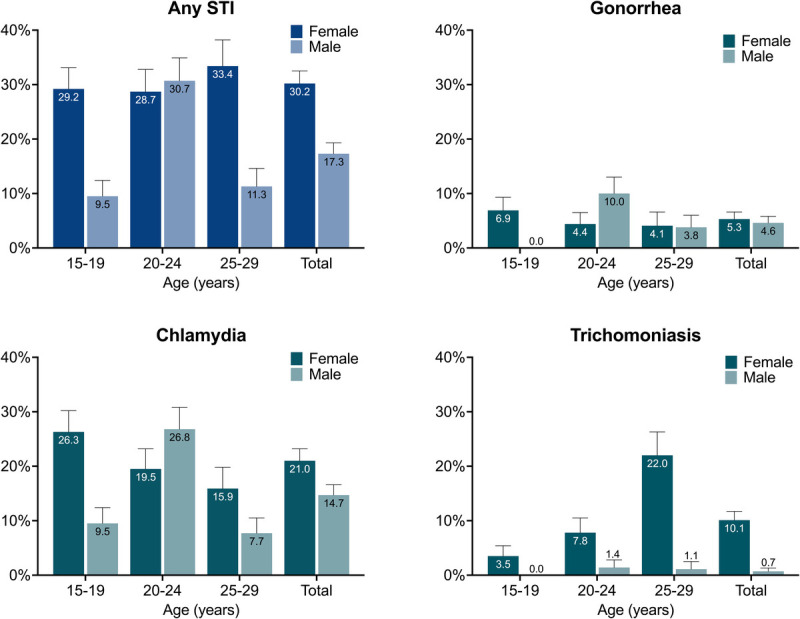
Population-weighted prevalence estimates for any STI and each individual STI, by sex and age group (with 95% CI).

Among the 814 specimens provided by participants, 14 of 814 (1.7%) had results of invalid or error for gonorrhea and chlamydia; of these, 3 (0.4%) also had invalid results for trichomoniasis. Of 800 participants with valid test results for all 3 STIs, 179 (22.4%) tested positive for at least 1 STI. Of these, 147 (82.1%) were monoinfections, whereas 32 participants (17.9%) were coinfected with more than 1 STI, including 3 participants (1.7%, all female) infected with 3 STIs concurrently (Supplemental Digital Content Table S1, http://links.lww.com/OLQ/A990). Population-weighted prevalence estimates for any STI and each STI individually, by sex and age group, are shown in Figure 2 and Supplemental Digital Content Table S2 (http://links.lww.com/OLQ/A991), demonstrating 30.2% prevalence of any STI among female participants and 17.3% prevalence among male participants.

In unadjusted analyses, STIs were more common among women, among those older than 20 years than 15 to 19 years, and those with urban/periurban compared with rural residence. Sexually transmitted infections were less common among those with unknown employment or marital status (who are also more likely to be <18 years old), and those with migration in the preceding 2 years. In adjusted analyses, STIs remained more than twice as likely among women than men (adjusted odds ratio [aOR], 2.14; 95% CI, 1.48–3.09; *P* = 0.0001), more likely among those residing in urban/periurban areas (aOR, 1.48; 95% CI, 1.02–2.15; *P* = 0.041; Table 2), and less likely among those with any recent migration (aOR, 0.37; 95% CI, 0.15–0.89; *P* = 0.026).

**TABLE 2:** Factors Associated With Diagnosis of Any STI (Chlamydia, Gonorrhea, or Trichomoniasis)

Demographic factor	No. With Any Curable STI, n/N (%)	Unadjusted OR (95% CI)	Age- and Sex-Adjusted OR (95% CI)	Multivariable analysis (n = 743), aOR
Age group (n = 800), y		*P* = 0.004	*P* = 0.0062	*P* = 0.08
16–19	48/291 (16.5)	1	1	1
20–24	76/272 (27.9)	1.96 (1.31–2.95)	1.96 (1.29–2.95)	1.72 (0.87–3.40)
25–29	55/237 (23.2)	1.53 (0.99–2.36)	1.45 (0.94–2.25)	1.10 (0.53–2.30)
Sex (n = 800)		*P* < 0.0001	*P* < 0.0001	***P* = 0.0001**
Male	60/386 (15.5)	1	1	**1**
Female	119/414 (28.7)	2.19 (1.55–3.10)	2.18 (1.54–3.10)	**2.14 (1.48–3.09)**
Education completed (n = 704)		*P* = 0.49	*P* = 0.74	
Some primary	7/27 (25.9)	1	1	
Some secondary	80/390 (20.5)	0.74 (0.30–1.80)	0.71 (0.28–1.76)	
Matric or above	69/287 (24.0)	0.90 (0.37–2.23)	0.69 (0.26–1.81)	
Employment (n = 800)		*P* = 0.024	*P* = 0.12	
Unemployed	107/444 (24.1)	1	1	
Employed*	20/63 (31.8)	1.46 (0.83–2.60)	1.84 (1.01–3.37)	
Unknown	52/293 (17.8)	0.68 (0.47–0.98)	1.30 (0.61–2.80)	
Marital status (n = 800)		*P* = 0.013	*P* = 0.90	*P* = 0.93
Not married	52/218 (23.9)	1	1	1
Married or informal union	88/339 (26.0)	1.12 (0.75–1.66)	1.01 (0.65–1.57)	1.07 (0.68–1.68)
Unknown	39/243 (16.1)	0.61 (0.38–0.97)	0.86 (0.43–1.70)	0.93 (0.46–1.88)
Migration in past 2 y (n = 745)^†^		*P* = 0.013	*P* = 0.017	***P* = 0.026**
Never	164/688 (23.8)	1	1	**1**
Any migration	6/57 (10.5)	0.38 (0.16–0.89)	0.34 (0.14–0.82)	**0.37 (0.15–0.89)**
Residence (n = 798)		*P* = 0.0022	*P* = 0.0069	***P* = 0.041**
Rural	111/569 (19.5)	1	1	**1**
Urban or periurban	68/229 (29.7)	1.74 (1.23–2.48)	1.64 (1.15–2.36)	**1.48 (1.02–2.15)**
SE status tertile (n = 764)		*P* = 0.36	*P* = 0.61	
Low	64/255 (25.1)	1	1	
Medium	51/254 (20.1)	0.75 (0.49–1.14)	0.81 (0.53–1.24)	
High	54/255 (21.2)	0.80 (0.53–1.21)	0.89 (0.58–1.36)	

Bold text indicates factors with *P*-value <0.05 in multivariable analyses.

*Includes full-time and part time employed.

^†^Any migration includes internal and external migration.

SE indicates socioeconomic.

Among participants with a positive STI result and complete follow-up data, 53 of 171 (31.0%) were treated within 7 days of specimen collection. Median time to treatment overall was 11 days (IQR, 6–77 days) and did not differ by sex or age group (data not shown). Among 73 participants not treated within 4 weeks of specimen collection, 51 (69.9%) could not be reached sooner and were treated later, 11 (15.1%) could not be contacted after multiple attempts, 6 (8.2%) had migrated outside of the area, and 5 (6.8%) refused treatment (reasons for refusal not provided). In analyses adjusted for age and sex, urban/periurban residence was associated with a lower likelihood of treatment within 7 days compared with rural residence (aOR, 0.42; 95% CI, 0.20–0.87; *P* = 0.019), whereas being in the highest socioeconomic tertile was associated with a higher likelihood of treatment within 7 days (aOR, 3.12; 95% CI, 1.36–7.16; *P* = 0.0032; Table 3).

**TABLE 3:** Factors Associated With STI Treatment (Within 7 days; n = 171)

	Treated Within 7 d, n/N (%)	Unadjusted OR (95% CI)	Age- and Sex-Adjusted OR (95% CI)
Age group, y		*P* = 0.32	*P* = 0.33
15–19	13/48 (27)	1	1
20–24	20/72 (28)	1.04 (0.46–2.35)	1.09 (0.47–2.53)
25–29	20/51 (39)	1.74 (0.74–4.06)	1.78 (0.76–4.17)
Sex		*P* = 0.56	*P* = 0.59
Male	16/57 (28)	1	1
Female	37/114 (32)	1.23 (0.61–2.48)	1.22 (0.59–2.51)
Education completed (n = 149)		*P* = 0.057	*P* = 0.18
Primary or less	1/7 (14)	1	1
Some secondary	19/77 (25)	1.97 (0.22–17.37)	2.15 (0.24–19.21)
Matric or above	27/65 (42)	4.26 (0.48–37.48)	4.69 (0.49–44.67)
Employment		*P* = 0.46	*P* = 0.44
Unemployed	35/101 (35)	1	1
Employed*	5/20 (25)	0.63 (0.21–1.87)	0.54 (0.17–1.69)
Unknown	13/50 (26)	0.66 (0.31–1.41)	0.57 (0.14–2.36)
Marital status		*P* = 0.016	*P* = 0.053
Not married	9/49 (18)	1	1
Married or informal union	34/83 (41)	3.08 (1.32–7.18)	3.06 (1.22–7.67)
Unknown	10/39 (26)	1.53 (0.55–4.25)	1.12 (0.27–4.73)
Migration in past 2 y (n = 167)		*P* = 0.93	*P* = 0.83
Never	51/161 (32)	1	1
Any migration^†^	2/6 (33)	1.08 (0.19–6.08)	1.22 (0.21–7.18)
Residence		*P* = 0.026	***P* = 0.019**
Rural	39/105 (37)	1	**1**
Urban or periurban	14/66 (21)	0.46 (0.22–0.93)	**0.42 (0.20–0.87)**
SE status tertile (n = 163)		*P* = 0.0086	***P* = 0.0032**
Low	17/61 (28)	1	**1**
Medium	10/48 (21)	0.68 (0.28–1.66)	**0.76 (0.30–1.89)**
High	26/54 (48)	2.40 (1.11–5.21)	**3.12 (1.36–7.16)**

Bold text indicates factors with *P*-value <0.05 in multivariable analyses.

*Includes full-time and part-time employment.

^†^Any migration includes internal and external migration.

SE indicates socioeconomic.

## DISCUSSION

We found a very high prevalence of curable STIs among adolescents and young adults in a predominantly rural area of KwaZulu-Natal, South Africa. This study confirms the acceptability of home-based STI specimen collection among adolescents and young adults, as more than 90% of study participants who were offered STI testing provided specimens. The prevalence of STI was significantly higher among female than male participants overall, even when adjusted for age and other demographic factors. The sex difference in prevalence was most pronounced for trichomoniasis and chlamydia; prevalence of gonorrhea was similar between male and female participants. Participants residing in urban/periurban areas were more likely to have an STI than those residing in rural areas. Despite multiple contact attempts by study staff, only 1 of 3 participants who tested positive for an STI was treated within 7 days. Difficulties in follow-up contact compounded by a low (6.8%) treatment refusal indicates a need for a robust tracking system and strategies to maximize treatment reach, and underscores the need for point-of-care STI tests to enable same-day treatment and decrease loss to follow-up.

In this cohort, women had a higher STI prevalence than men overall, particularly trichomoniasis and chlamydia. These results mirror both national-level estimates of STI prevalence in South Africa and previous studies among adolescents and young adults in rural KwaZulu-Natal; in both cases, chlamydia prevalence was over twice as high among women than men.^13,15^ Young women in South Africa may face a higher risk of STI acquisition than age-matched male counterparts because of earlier age of sexual debut,^15^ higher rate of age-disparate relationships,^20^ and lesser ability to navigate safe sex. Gender inequalities contribute to the higher rates of STIs among women than men in many parts of the world, and adolescent girls and young women have been identified as priority populations for STI programming by the WHO.^11^ Furthermore, because STIs are more often asymptomatic in women than men, fewer women may receive treatment through syndromic management pathways, leading to longer duration of infection and thus detection of a greater prevalence of active infections among women. However, we found that among those aged 20 to 24 years, men had a higher prevalence of gonorrhea and chlamydia than women. A previous study in this setting also found a higher prevalence of chlamydia among men than women in this age group (12.2% vs. 10.6%, respectively).^15^ Reasons for this finding are not clear but may relate to later sexual debut among men in this setting.^15^ Differences in sexual networks, transactional sex, or migration may also contribute to this finding; however, because of limited data on young men, it is difficult to know which factors account for it. This is, however, an important observation that requires further study.

We found a substantially higher prevalence of chlamydia and gonorrhea in this cohort than in a previous study conducted in the same geographic area in 2016 to 2017.^15^ Weighted prevalence estimates for chlamydia were 8.1% in the previous study and 17.9% in the current study, and for gonorrhea, the values were 1.7% in the prior study and 4.6% in the current study. The previous study enrolled adolescents and young adults up to age 25, whereas the current study enrolled adults up to age 29 years’ however, prevalence estimates were higher in the current study within each individual age group and overall, with the exception of trichomoniasis in men. The high STI prevalence estimates for women in the current study mirror emerging data on STI prevalence among women enrolled in preexposure prophylaxis trials and women living with HIV in Southern Africa.^21–23^ The difference in prevalence estimates between this and the previous study may thus signal an increase in STIs over time in this area, supporting an urgent need for greater access to sexual health services for this population. Furthermore, nonpharmaceutical interventions adopted during the COVID-19 pandemic, such as national lockdowns, could have impacted transmission within sexual networks, contributing to the higher STI prevalence found in this study.

We additionally found that young men and women residing in urban or periurban areas were more likely to have an STI than those residing in rural areas, even after adjustment for other demographic factors, including age, employment status, and migration history. A recent study evaluating transmissible HIV among adolescent girls and young women exposed to the PEPFAR-supported DREAMS intervention, conducted at the same study site, similarly found that urban/periurban residence was associated with transmissible HIV.^24^ HIV incidence over time was higher in urban and periurban areas of the study site in a separate study.^25^ Despite the predominantly rural nature of the AHRI HDSS area, there are several informal periurban settlements and an urban township with high population density.^26^ Potential differences between the urban/periurban and rural participants, such as differences in socioeconomic status, substance use, transactional sex, gender-based violence, patterns of sexual behavior, or migration history, may explain the difference in STI prevalence. In addition, greater movement of people through the urban areas may contribute to higher turnover of partners and lead to more introduction of infections into the community; however, more study of potential drivers is needed. We additionally found that adolescents and young adults reporting recent migration had lower odds of having an STI than those who had not migrated in the same period. Although this finding could reflect higher STI transmission in local sexual networks, the small number of participants with recent migration events makes it difficult to draw conclusions from this finding. Additional data on sexual risk behavior obtained at the end point of the trial may help elucidate the reasons behind these observed differences.

Despite the robust infrastructure of the randomized trial and the long-standing experience of AHRI conducting research that is strongly linked with public sector health clinics in this area, less than half of the participants with STIs were able to be treated within 7 days, and less than two-thirds within 4 weeks. Those living in urban areas were less likely to be treated within 7 days, possibly because of a higher rate of employment or difficulty tracking participants. Those in the highest socioeconomic tertile were more likely to be treated within 7 days, which may reflect either greater access to technology such as mobile phones for contact by study staff, or easier access to clinic for treatment. Diagnostic testing for STIs remains inaccessible in most resource-limited settings, because of high costs and a need for laboratory infrastructure; when STI testing is available in such settings, it is often restricted to centralized laboratories. For this study, STI specimens were transported from the rural study site to a centralized research laboratory in Durban (approximately 230 km away), resulting in an extended time from specimen collection to test result. Loss to follow-up increases with extensions in test turnaround time, and delays in treatment lead to the potential for ongoing transmission and increased risk for sequelae of untreated infection. A study assessing community-based STI testing for adolescents and young adults in Zimbabwe found that, even with an expected 90-minute time to result, only 67% of those with positive test results were treated.^27^ These findings highlight the urgency of development and implementation of affordable point-of-care STI diagnostics that meet the WHO REASSURED criteria (Real-time connectivity, Ease of specimen collection, Affordable, Sensitive, Specific, User-friendly, Rapid and robust, Equipment free or simple and Environmentally friendly, Deliverable to end-users)^28^ and enable immediate treatment and partner notification services.

Our assessment of factors associated with STIs was limited by the scope of demographic data available and lack of contemporaneous data on symptoms and sexual risk behavior. The trial did not include study-specific questionnaires at the time of enrollment to measure the real-world effect of offering the combination of interventions. Demographics were thus linked from annual HDSS household surveys. These surveys include annually updated, individual- (e.g., education level, employment status) and household-level (e.g., socioeconomic status, rural vs. urban residence) data. Despite a lack of detail regarding sexual risk behavior, the HDSS data provide information on several important demographics that are standardized across prior studies and have previously been found to be associated with STIs and HIV in this area.^15,24,25,29^ We were also unable to assess the prevalence of STI symptoms; however, a previous study in this area found that 75% of female participants with an STI were asymptomatic.^15^ In addition, concurrent HIV testing was not conducted, as linkage to HIV testing was part of the primary outcome of the randomized controlled trial. Thus, STI prevalence in this cohort cannot be stratified by HIV status; however, other studies have found a higher prevalence of curable STIs among people living with HIV than those without HIV, particularly among women.^23^ Furthermore, approximately one-quarter of female participants provided urine specimens, which have a slightly lower sensitivity than vaginal swab specimens,^30^ and may have led to an underestimation of STI prevalence among female participants. Finally, several participants had invalid STI test results; however, this was a small percentage of the total cohort (<2%). The use of point-of-care tests in future surveillance or clinical settings could allow for the collection of a repeat specimen if a first is found to be inadequate or does not pass an internal control.

In conclusion, we found a very high prevalence of curable STIs among adolescent and young adult men and women, which is higher than in a previous study 5 years ago, in a predominantly rural area with high HIV incidence in KwaZulu-Natal, South Africa. The prevalence of STI was higher among women than men and among those residing in urban/periurban areas than those residing in rural areas. Despite multiple attempts by study staff, fewer than two-thirds of participants with positive test results were able to be treated within 4 weeks. These results highlight the need for implementation of STI testing and treatment programs in settings with both STIs and HIV, as well as the need for point-of-care STI tests to allow immediate treatment for those who test positive and decrease loss to follow-up.

## Supplementary Material

SUPPLEMENTARY MATERIAL
