# Supplementary material for: Prevalence of Curable Sexually Transmitted Infections in a Population-Representative Sample of Young Adults in a High HIV Incidence Area in South Africa
Source: Sex Transm Dis. 2023 Oct 8;50(12):796–803. doi: 10.1097/OLQ.0000000000001871 (PMC10655853; doi:10.1097/OLQ.0000000000001871)
Supplement: SUPPLEMENTARY MATERIAL [file std-50-796-s001.docx]

**Jarolimova J et al.** **Prevalence of curable sexually transmitted infections in a population-representative sample of young adults in a high HIV incidence area in South Africa**

**Supplemental Table S1. Unweighted prevalence of STIs by age and sex.** Among participants with valid test results. All data presented as n/N (%, 95% CI).

|  | Female, age 16-19 | Female, age 20-24 | Female, age 25-29 | Female overall | Male, age 16-19 | Male, age 20-24 | Male, age 25-29 | Male overall | Total |
| --- | --- | --- | --- | --- | --- | --- | --- | --- | --- |
| Any STI; n=800 | 39/141 (27.7, 20.9-35.6) | 41/139 (29.5, 22.5-37.6) | 39/134 (29.1, 22.0-37.4) | 119/414 (28.7, 24.6-33.3) | 9/150 (6.0, 3.1-11.1) | 35/133 (26.3, 19.5-34.5) | 16/103 (15.5, 9.7-23.9) | 60/386 (15.5, 12.3-19.5) | 179/800 (22.4, 19.6-25.4) |
| Total gonorrhea, n=800 | 10/141 (7.1, 3.9-12.7) | 9/139 (6.5, 3.4-12.0) | 5/134 (3.7, 1.6-8.7) | 24/414 (5.8, 3.9-8.5) | 0/150 | 9/133 (6.8, 3.6-12.5) | 4/103 (3.9, 1.5-9.9) | 13/386 (3.4, 2.0-5.7) | 37/800 (4.6, 3.4-6.3) |
| Total chlamydia, n=800 | 34/141 (24.1, 17.8-31.9) | 32/139 (23.0, 16.8-30.8) | 24/134 (17.9, 12.3-25.3) | 90/414 (21.7, 18.0-26.0) | 9/150 (6.0, 3.1-11.1) | 33/133 (24.8, 18.2-32.9) | 11/103 (10.7, 6.0-18.3) | 53/386 (13.7, 10.6-17.5) | 143/800 (17.9, 15.4-20.7) |
| Total trichomoniasis, n=811 | 6/142 (4.2, 1.9-9.1) | 7/147 (4.8, 2.3-9.7) | 20/136 (14.7, 9.7-21.7) | 33/425 (7.8, 5.6-10.7) | 0/150 | 0/133 | 2/103 (1.9, 0.5-7.4) | 2/386 (0.5, 0.1-2.1) | 35/811 (4.3, 3.1-5.9) |
| Gonorrhea mono-infection, n=800 | 1/141 (0.7) | 2/139 (1.4) | 2/134 (1.5) | 5/414 (1.2) | 0/150 | 2/133 (1.5) | 4/103 (3.9) | 6/386 (1.6) | 11/800 (1.4) |
| Chlamydia mono-infection, n=800 | 24/141 (17.0) | 27/139 (19.4) | 17/134 (12.7) | 68/414 (16.4) | 9/150 (6.0) | 26/133 (19.6) | 10/103 (9.7) | 45/386 (11.7) | 113/800 (14.1) |
| Trichomoniasis mono-infection, n=800 | 4/141 (2.8) | 5/139 (3.6) | 13/134 (9.7) | 22/414 (5.3) | 0/150 | 0/133 | 1/103 (1.0) | 1/386 (0.26) | 23/800 (2.9) |
| Gonorrhea-chlamydia coinfection, n=800 | 8/141 (5.7) | 5/139 (3.6) | 1/134 (0.8) | 14/414 (3.4) | 0/150 | 7/133 (5.3) | 0/103 | 7/386 (1.8) | 21/800 (2.6) |
| Chlamydia-trichomoniasis coinfection, n=800 | 1/141 (0.7) | 0/139 | 4/134 (3.0) | 5/414 (1.2) | 0/150 | 0/133 | 1/103 (1.0) | 1/386 (0.3) | 6/800 (0.8) |
| Gonorrhea-trichomoniasis coinfection, n=800 | 0/141 | 2/139 (1.4) | 0/134 | 2/414 (0.5) | 0/150 | 0/133 | 0/103 | 0/386 | 2/800 (0.3) |
| Gonorrhea-chlamydia-trichomoniasis coinfection, n=800 | 1/141 (0.7) | 0/139 | 2/134 (1.5) | 3/414 (0.7) | 0/150 | 0/133 | 0/104 | 0/387 | 3/800 (0.4) |
| Among STI positive, received treatment (have recorded treatment date within 4 weeks of STI test result), n=174 | 24/39 (62) | 24/40 (60) | 23/36 (64) | 71/115 (62) | 5/9 (56) | 18/35 (51) | 4/15 (27) | 27/59 (46) | 98/174 (56) |
