# Supplementary material for: Prevalence of Curable Sexually Transmitted Infections in a Population-Representative Sample of Young Adults in a High HIV Incidence Area in South Africa
Source: Sex Transm Dis. 2023 Oct 8;50(12):796–803. doi: 10.1097/OLQ.0000000000001871 (PMC10655853; doi:10.1097/OLQ.0000000000001871)
Supplement: SUPPLEMENTARY MATERIAL [file std-50-796-s002.docx]

**Jarolimova J et al.** **Prevalence of curable sexually transmitted infections in a population-representative sample of young adults in a high HIV incidence area in South Africa**

**Supplemental Table S2. Weighted prevalence estimates for STIs overall and by sex and age category.** All data presented as % (95% CI).

|  | Female, age 16-19 | Female, age 20-24 | Female, age 25-29 | Female overall | Male, age 16-19 | Male, age 20-24 | Male, age 25-29 | Male overall | Total |
| --- | --- | --- | --- | --- | --- | --- | --- | --- | --- |
| Any STI | 29.2 (25.4 -33.1) | 28.7 (24.8-32.8) | 33.4 (28.9-38.2) | 30.2 (27.8 -32.5) | 9.5 (7.2-12.4) | 30.7 (26.7-34.9) | 11.3 (8.6-14.6) | 17.3 (15.4-19.3) | 23.7 (22.2-25.3) |
| Gonorrhea | 6.9 (4.9-9.3) | 4.4 (2.8-6.5) | 4.1 (2.4-6.6) | 5.3 (4.3-6.6) | 0 | 10.0 (7.6-13.0) | 3.8 (2.3-6.0) | 4.6 (3.6-5.8) | 5.0 (4.2-5.8) |
| Chlamydia | 26.3 (22.7-30.2) | 19.5 (16.2-23.2) | 15.9 (12.5-19.8) | 21.0 (19.0-23.2) | 9.5 (7.2-12.4) | 26.8 (23.0-30.8) | 7.7 (5.4-10.5) | 14.7 (13.0 -16.6) | 17.9 (16.5-19.3) |
| Trichomoniasis | 3.5 (2.2-5.4) | 7.8 (5.7-10.5) | 22.0 (18.0-26.3) | 10.1 (8.6-11.7) | 0 | 1.4 (0.6-2.8) | 1.1 (0.3-2.5) | 0.7 (0.4-1.3) | 5.4 (4.6-6.3) |
